# Molecular dynamics simulation of the interaction between palmitic acid and high pressure CO_2_

**DOI:** 10.1098/rsos.231141

**Published:** 2023-11-22

**Authors:** Fei Li, Fayu Sun, Zirui Li, Zihao Zheng, Weiqiang Wang

**Affiliations:** Key Laboratory of High-efficiency and Clean Mechanical Manufacture (Ministry of Education), National Demonstration Center for Experimental Mechanical Engineering Education (Shandong University), Research Center for Sustainable Manufacturing (Shandong University), School of Mechanical Engineering, Shandong University, Jinan 250061, Shandong, People's Republic of China

**Keywords:** palmitic acid, carbon dioxide, molecular simulation, interaction

## Abstract

In this study, molecular dynamics simulation was used to explore the interaction characteristics of palmitic acid and CO_2_, and the effects of temperature and pressure on the solubility of palmitic acid in CO_2_ were investigated. In the range of 293–353 K and 5–30 MPa, the snapshot of palmitic acid distribution in CO_2_ shows that the molecular chain of palmitic acid in high-density CO_2_ system is more straight and more dispersed than that in low-density CO_2_ system. The radial distribution function further clearly shows that the solubility of palmitic acid in CO_2_ decreases with the increase of temperature and increases with the increase of pressure, which is consistent with the fatty acid solubility data reported in the literature and the setting rules of supercritical CO_2_ extraction process conditions. As the temperature decreases and the pressure increases, the interaction energy between palmitic acid and CO_2_ increases, which is conducive to overcoming the intermolecular force of palmitic acid and promoting dissolution. The solubility parameters of palmitic acid and CO_2_ can better reflect the trend of palmitic acid solubility changing with temperature and pressure, which can play a guiding role in the determination of process conditions and even the development of new processes.

## 1. Introduction

In recent years, the supercritical fluid technology, as a green technology, has received great attention from the academic community [1,2]. It has made great research progress in extraction, pharmacy, dyeing, foaming, spraying and other fields [3,4], and has been widely used in oil extraction [5]. Carbon dioxide is the most widely used solvent in supercritical fluid extraction technology because of its mild conditions, non-toxic, harmless, easy separation and no residue [6].

At present, using experimental methods to study the effects of supercritical carbon dioxide extraction process conditions, such as temperature, pressure, etc., on oil extraction rate and different components, especially for new experimental materials, is still the current research hotspot. Lasekan & Abdulkarim [7] used supercritical carbon dioxide to extract oil from tiger nuts, and studied the effect of temperature (40–80°C), pressure (20–40 MPa) and time (60–360 min) on oil yield. Moreover, the fatty acid composition of oils extracted by supercritical carbon dioxide and Soxhlet showed significant changes. Duba *et al*. [8] measured the solubility of grape seed oil in supercritical carbon dioxide at temperatures ranging from 313 to 343 K and pressures ranging from 20 to 50 MPa, and conducted thermodynamic modelling of solubility. Vasquez *et al*. [9] carried out supercritical CO_2_ extraction to recover the lipid part of cake by-product from Brazil nut beverage, and evaluated the effects of pressure and temperature on the yield, oil recovery rate, fatty acid distribution and small amounts of lipid compounds. Mouahid *et al*. [10] investigated the effects of process parameters such as pressure (20–40 MPa), temperature (313–333 K) and flow rate (0.11–0.27 kg h^−1^) on the efficiency of supercritical CO_2_ extraction of Argan oil using response surface methodology. On the whole, the effects of temperature and pressure on oil extraction rate and component selectivity can be largely attributed to the interaction between fatty acids and high-pressure CO_2_, as the composition of oil is the esterification of fatty acids. Although some scholars have studied the solubility data of fatty acids in supercritical carbon dioxide for a long time, the data are relatively limited, and the solubility data of fatty acids in liquid carbon dioxide near the critical region is even more scarce [11,12]. Moreover, studies such as extraction kinetics experiments and solubility experiments only examined the impact of process conditions at the macro level, without explaining the micro dissolution mechanism.

Molecular dynamics (MD) simulation technology can complete the characterization and analysis of molecular characteristics that cannot be achieved at the experimental level, intuitively providing information on the conformational changes of molecules during the simulation process and the interactions between different components, thus providing strong theoretical support for microscopic mechanism analysis. As an important means of studying micro mechanisms, MD simulation has been widely applied in fields such as chemical engineering, materials science and engineering, and biomedicine [13–16].

In terms of chemical composition, oils are esters formed by advanced fatty acids and glycerol. Glycerol esters are esters formed by the esterification of one molecule of glycerol and three molecules of fatty acids, also known as triglycerides. The properties of glycerol esters mainly depend on the properties of the fatty acids in the composition. According to the different saturations of C-H bonds, fatty acids are divided into saturated fat fatty acids and unsaturated fat fatty acids. According to the different length of carbon chain, it can be divided into short-chain fatty acid (with the number of carbon atoms less than six), medium-chain fatty acids (with the number of carbon atoms more than six but less than 12) and long-chain fatty acids (with the number of carbon atoms more than 12). Most common fatty acids in daily life are medium-chain and long-chain fatty acids, such as oleic acid, linoleic acid, palmitic acid, etc. Different fatty acids participate in different metabolism and play different physiological functions in the human body, and selective intake is of great significance for regulating physical health [17,18]. Although fatty acids can be divided into many categories, they have the same molecular structure and are composed of carbon chains with methyl groups at one end and carboxyl groups at the other. Palmitic acid is a kind of advanced saturated fatty acid, which widely exists in nature. Almost all oils contain palmitic acid in varying amounts. The content of palmitic acid in palm oil is approximately 40%, and that in Chinese tallow oil can be up to more than 60%.

In this study, palmitic acid was taken as a typical example, and the interaction between palmitic acid and high pressure carbon dioxide was studied from a microscopic perspective by means of molecular dynamics simulation. The microscopic mechanism of fatty acid dissolution was analysed in depth, providing theoretical support for determining and optimizing process conditions of super/subcritical carbon dioxide extraction, and providing new solutions for the application of super/subcritical fluid technology in fatty acid-related products beyond experiments.

## 2. Computational details

All simulations in this study were calculated using the Amorphous Cell and Forcite modules of Materials Studio 2017 (Accelrys, San Diego, CA, USA). The force field parameters of CO_2_ and fatty acids are both based on condensed phase optimized molecular potential for atomic simulation studies (COMPASS), which is an *ab initio* force field widely applicable to organic and inorganic material systems [19,20].The total potential energy *E*_total_ includes bonding energy and non-bonding energy, and the function expression is as follows:

2.1 $$E_{\mathrm{total}}=E_{\mathrm{bond}}+E_{\mathrm{angle}}+E_{\mathrm{torsion}}+E_{\mathrm{oop}}+E_{\mathrm{cross}}+E_{\mathrm{vdW}}+E_{\mathrm{coulombic}}.$$

In equation (2.1), *E*_bond_, *E*_angle_, *E*_torsion_, *E*_oop_, *E*_cross_, *E*_vdW_, *E*_coulombic_, respectively, represent the energy components of bond stretching, angular bending, dihedral angle torsion, out of plane interaction and cross coupling, short distance van der Waals interaction, and long distance electrostatic interaction. The first five terms form the bonding potential, while the last two terms form the non-bonding potential. The vdW interaction is represented by the Lennard–Jones 96 function, while the electrostatic interaction is calculated by the Coulomb equation and described by the following equations, respectively.

2.2 $$E_{\mathrm{vdW}}=D_{0}\left[2{\left(\frac{R_{0}}{R}\right)}^{9}-3{\left(\frac{R_{0}}{R}\right)}^{6}\right]$$

and

2.3 $$E_{\mathrm{coulombic}}=C\frac{q_{i}q_{j}}{\varepsilon R}.$$

In equations (2.2) and (2.3), C = 332.0647 (kcal mol^−1^) Å e^−2^ is the unit conversion factor, *D*_0_ is the depth of the potential well, *q_i_* and *q_j_* are partial charges of atoms *i* and *j*, respectively, *R* is the distance between atoms, *R*_0_ is the Lennard–Jones radius, and *ε* is the relative dielectric constant.

**Figure 1.**
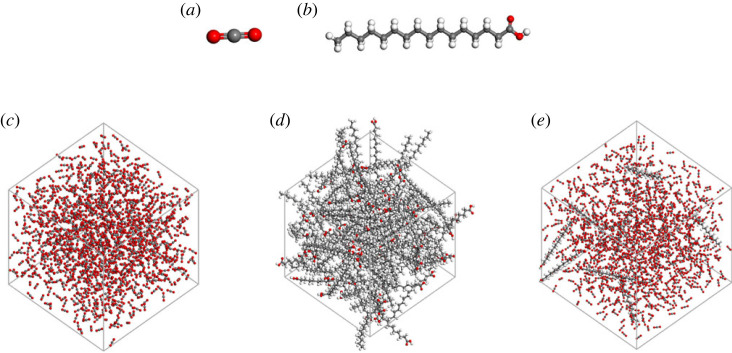
Initial models: (*a*) CO_2_ molecule, (*b*) palmitic acid molecule, (*c*) cell containing 1000 pure CO_2_ molecules, (*d*) cell containing 100 pure palmitic acid molecules, (*e*) cell containing eight palmitic acid molecules and 1000 CO_2_ molecules.

Firstly, palmitic acid molecule and CO_2_ molecule were established and their structures were optimized. Then, three cells containing 1000 pure CO_2_ molecules, 100 pure palmitic acid molecules, eight palmitic acid molecules and a mixture of 1000 CO_2_ molecules were established, as shown in figure 1. The solubility of palmitic acid in high pressure CO_2_ can reach an order of magnitude of 1% by mass fraction. The mass ratio of eight palmitic acid molecules to 1000 CO_2_ molecules is within this range, making modelling closer to the actual state and easier to observe and analyse the dispersion and aggregation state of palmitic acid in CO_2_. Although the system only contained approximately 3000 atoms, under the action of periodic boundary conditions, the accuracy of the calculation was ensured and the speed of the calculation was achieved. The initial density of the 1000 pure CO_2_ molecules system, eight palmitic acid molecules and 1000 CO_2_ molecules mixture system was established based on the actual density of CO_2_ in the National Institute of Standards and Technology (NIST) database at that temperature and pressure. And the 100 pure palmitic acid molecules system was established based on the actual palmitic acid density of 0.852 g cm^−3^.

**Figure 2.**
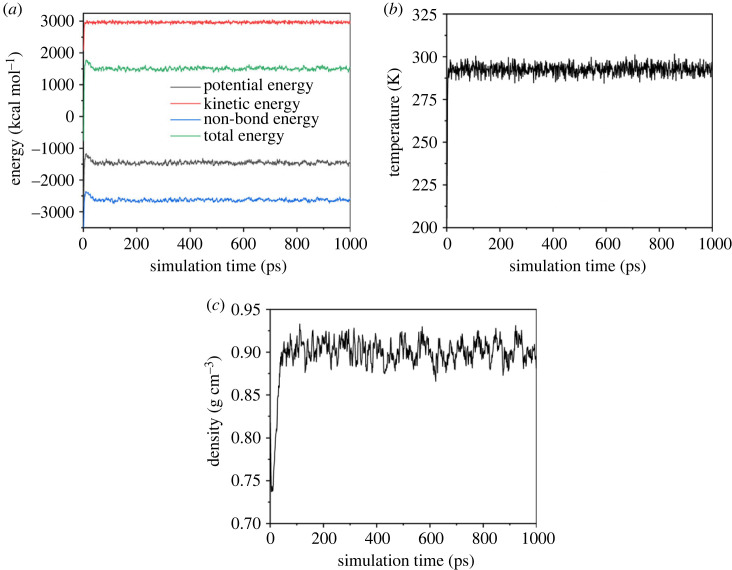
(*a*) Energy, (*b*) temperature, (*c*) density fluctuations observed during molecular dynamic simulation.

Then, the smart algorithm [21] was used to optimize the geometry for 50 000 steps. The convergence of energy was 2.0 × 10^−5^ kcal mol^−1^, the convergence of force was 0.001 kcal mol Å^−1^, and the convergence of displacement was 1.0 × 10^−5^ Å. Finally, the molecular dynamics equilibrium of NPT ensemble was carried out under the conditions of 293–353 K and 5–30 MPa in a time step of 1 fs. Considering the saving of computing resources and good conservation of energy, the integrator adopted the Verlet velocity algorithm. Andersen temperature control method and Berendsen pressure control method were used. The electrostatics was calculated by Ewald method with Ewald accuracy of 1.0 × 10^−5^ kcal mol^−1^, while van der Waals was calculated by atom-based method with cut-off distance of 18.5 Å. In general, the system reaches equilibrium when the standard deviation of the energy, temperature and density requirements of the system is less than 10%. At around 100 ps, both the density and energy have converged, indicating that the system has reached equilibrium. In order to obtain more accurate data results, the simulation process has been extended to 1000 ps. Taking the mixed system of eight palmitic acid molecules and 1000 CO_2_ molecules at 293 K and 10 MPa as an example, the energy, temperature and density fluctuations observed during molecular dynamic simulation are shown in figure 2. The last 500 ps was used for statistical analysis of solubility parameters, interaction energy and radial distribution function. And the influence of temperature and pressure on the solubility of palmitic acid was discussed based on the analysis results.

## 3. Results and discussion

### 3.1. Microscopic distribution of palmitic acid in CO_2_

**Figure 3.**
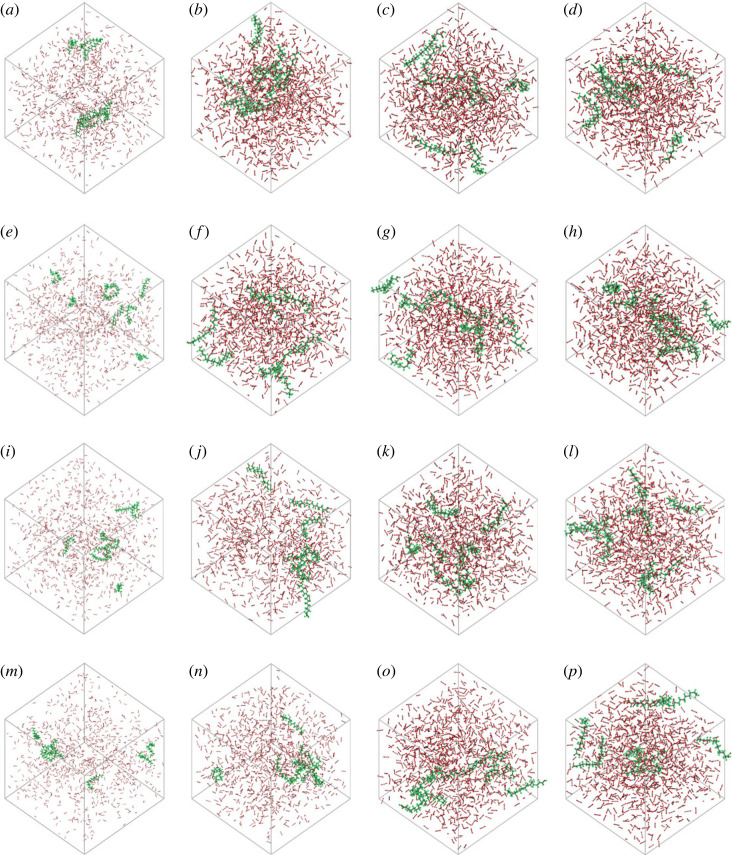
Microscopic distribution of palmitic acid in CO_2_: (*a*) 293 K 5 MPa, (*b*) 293 K 10 MPa, (*c*) 293 K 20 MPa, (*d*) 293 K 30 MPa, (*e*) 313 K 5 MPa, (*f*) 313 K 10 MPa, (*g*) 313 K 20 MPa, (*h*) 313 K 30 MPa, (*i*) 333 K 05 MPa, (*j*) 333 K 10 MPa, (*k*) 333 K 20 MPa, (*l*) 333 K 30 MPa, (*m*) 353 K 5 MPa, (*n*) 353 K 10 MPa, (*o*) 353 K 20 MPa, (*p*) 353 K 30 MPa.

The dissolution process of palmitic acid solid in high pressure CO_2_ can be considered as gradual diffusion of palmitic acid molecules from palmitic acid molecular group to surrounding CO_2_. If the actual situation is modelled, the reductive dissolution process can be more realistic to a certain extent. However, this study is more concerned about the interaction between palmitic acid and CO_2_ molecules after the system is balanced. Therefore, regardless of temperature and pressure conditions, Amorphous Cell was used to distribute eight palmitic acid molecules almost evenly in a cell filled with CO_2_, which can greatly save the computational resource consumption caused by the non-equilibrium process of dissolution to phase equilibrium. According to the different solubility of palmitic acid in high pressure CO_2_ under different temperature and pressure conditions, it can be determined that the system thus established is not a balanced real system. After deep equilibrium under 1000 ps NPT ensemble, fatty acid molecules show different degrees of dispersion in the cell. The distribution snapshot after equilibrium is shown in figure 3. Under different temperature and pressure conditions, the size of the box is different. Combined with the pure CO_2_ density, the solubility of palmitic acid under different temperature and pressure conditions can be judged visually.

With the help of the NIST Chemistry WebBook [22], the actual density data of CO_2_ can be easily obtained. At the same time, density analysis was conducted on pure CO_2_ systems under different temperature and pressure conditions after deep equilibrium. The simulation results and the actual density in the NIST Chemistry WebBook are shown in table 1. The minimal error between the molecular dynamics simulation results and the actual values also indicates the feasibility of the simulation method.

**Table 1.** Simulated density and actual density of pure CO_2_ (g cm^−3^).

T (K)	P (MPa)							
	5		10		20		30	
	MD	NIST	MD	NIST	MD	NIST	MD	NIST
293	0.137	0.141	0.878	0.857	0.957	0.938	1.000	0.985
313	0.114	0.113	0.708	0.632	0.860	0.841	0.926	0.910
333	0.999	0.098	0.290	0.291	0.746	0.725	0.850	0.830
353	0.090	0.088	0.223	0.222	0.623	0.595	0.766	0.746

From figure 3 and table 1, it can be seen that the molecular chain of palmitic acid is more straight and the molecules are more dispersed under the high CO_2_ density. By contrast, under the low CO_2_ density, palmitic acid is intertwined and aggregated. This also shows that the solubility of palmitic acid in high density CO_2_ is greater than that in low density CO_2_, which generally conforms to the basic general knowledge that the solubility of oil in high pressure CO_2_ increases with the increase of CO_2_ density and decreases with the decrease of CO_2_ density. When the system is large enough, the distribution of palmitic acid will be more clear, but it is limited by computing resources. In order to clarify the interaction characteristics of palmitic acid and CO_2_, quantitative analysis was carried out from the perspectives of radial distribution function, interaction energy, solubility parameters, etc.

### 3.2. Radial distribution function

**Figure 4.**
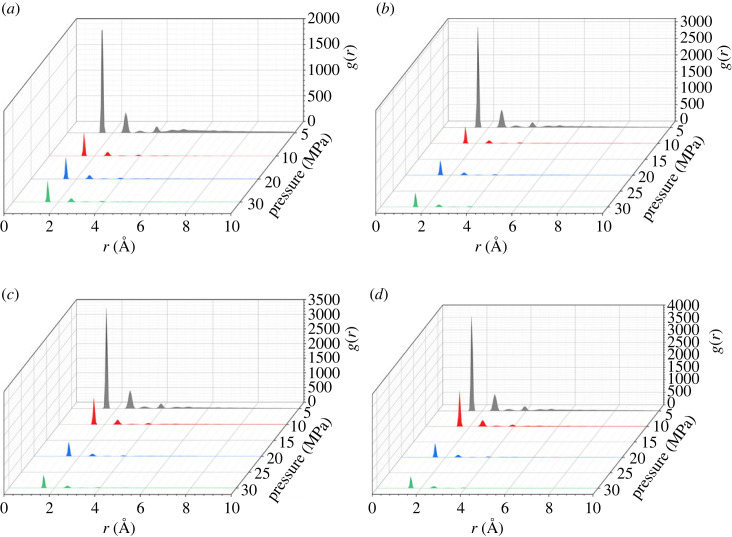
RDF curve between C atoms of palmitic acid after equilibrium: (*a*) 293 K, (*b*) 313 K, (*c*) 333 K, (*d*) 353 K.

The radial distribution function (RDF) is a description of the local distribution of other groups around a group in the shell. It refers to the ratio of the probability density of another particle at the distance *r* from a given particle to the average distribution probability density. The calculation of the radial distribution function *g(r)* is shown in equation (3.1). The location and size of the radial distribution function peak can reflect the variation of intermolecular bonding strength with distance, which means that the microscopic distribution details of palmitic acid can be linked with the macroscopic solubility. Figure 4 shows the RDF curve between C atoms of palmitic acid after equilibrium.

3.1 $$g(r)=\frac{dN}{4\rho \pi r^{2}dr}.$$

In equation (3.1), d*N* represents the number of group A between the distance *r* and *r* + d*r* from the reference group B, *ρ* represents the average number density of group A.

It can be seen from figure 4 that, under different temperatures and pressures, the positions of the first peaks of RDF remain unchanged, almost all at 1.525 Å, indicating that the distance between palmitic acid molecules to interact has not changed significantly. At the same temperature, with the increase of pressure, the peak value gradually decreases, which means that the aggregation degree of palmitic acid molecules decreases, indicating that the dispersion degree or solubility of palmitic acid increases with the increase of pressure. Under the same pressure, with the increase of temperature, the peak value gradually increases, which means that the aggregation degree of palmitic acid molecules increases, indicating that the dispersion degree or solubility of palmitic acid decreases with the increase of temperature. This is also consistent with the solubility data of fatty acids, oils and common supercritical CO_2_ extraction and separation process conditions [23,24]. However, existing molecular dynamics simulations and results only predict solubility trends and cannot accurately predict solubility. Generally, accurate prediction of solubility requires a detailed understanding of molecular interactions, which should also be an important focus of future research.

### 3.3. Interaction energy

In order to further clarify the interaction between palmitic acid and CO_2_, the interaction energy was calculated. The interaction energy can reflect the binding strength between palmitic acid and CO_2_. The larger the absolute value is, the stronger the interaction between the two substances will be, which means the better solubility of palmitic acid in CO_2_. Its expression is shown in the following equation:

3.2 $$E_{\mathrm{inter}}=E_{{\mathrm{CO}}_{2}+C16:0}-E_{{\mathrm{CO}}_{2}}-E_{C16:0},$$

where *E*_inter_ is the interaction energy between CO_2_ and palmitic acid, *E*_CO_2___+__C16__:__0_ is the total energy of the system, *E*_CO_2__ is the CO_2_ energy in the system, and *E*_C16__:__0_ is the palmitic acid energy in the system.

**Figure 5.**
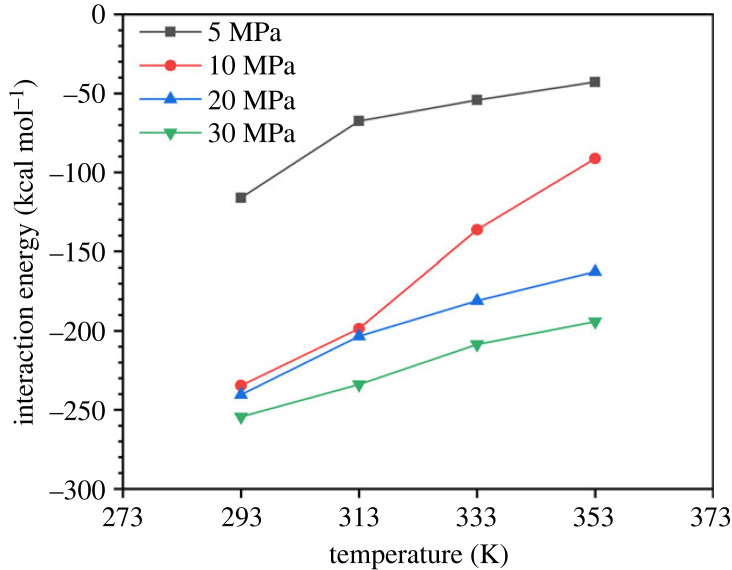
Interaction energy between palmitic acid and CO_2._

After deep equilibrium of the system, the interaction energy under various temperature and pressure conditions is shown in figure 5. At the same temperature, with the increase of pressure, the interaction energy increases, which means that the interaction between CO_2_ and fatty acids is strengthened. It is conducive to palmitic acid overcoming the binding between its own molecules and diffusing to the surrounding CO_2_, increasing its solubility in CO_2_. Under the same pressure, with the increase of temperature, the interaction energy decreases, which means that the interaction between CO_2_ and palmitic acid weakens. The binding between its own molecules is adverse to the diffusion of palmitic acid into CO_2_, thus reducing the solubility of palmitic acid in CO_2_. In the field of supercritical fluid extraction, this can be used to select lower temperature and higher pressure as the extraction process conditions. On the contrary, at high temperature and low pressure, the interaction energy is low and the solubility is poor, which can be used to achieve the separation of fatty acids and CO_2_.

### 3.4. Solubility parameter

The solubility parameter can reflect the solubility characteristics between different substances. In order to clearly characterize the solubility difference of palmitic acid in CO_2_ under different temperature and pressure conditions, the solubility parameters of palmitic acid and CO_2_ in the range of 293–353 K, 5–30 MPa were calculated by molecular dynamics method. In addition, in order to verify the correctness of molecular dynamics calculation, the group contribution method was used to calculate the solubility parameters of palmitic acid.

The solubility parameters of palmitic acid at normal temperature and pressure, 293–353 K, 5–30 MPa were calculated by molecular dynamics method. The results are shown in table 2. The changes in fatty acid solubility parameters caused by temperature and pressure can be almost negligible, so the solubility parameter 19.884 MPa^1/2^ at 298 K and 0.1 MPa will be used for subsequent analysis.

**Table 2.** Solubility parameters of palmitic acid (MPa^1/2^).

T (K)	P (MPa)				
	0.1	5	10	20	30
298	19.884	—	—	—	—
293	—	20.025	20.161	20.130	20.253
313	—	19.815	19.778	19.931	20.101
333	—	19.386	19.437	19.673	19.793
353	—	19.020	19.035	19.205	19.362

The group contribution method is the most widely used theoretical method to calculate the solubility parameters. Since Small first proposed calculating the solubility parameters based on the structure of substances in 1953 [25], a variety of group contribution methods have been developed, and their calculation results are similar. In this paper, Hoftyzer–Van Krevelen method [26] was used to calculate the solubility parameters of palmitic acid under normal temperature and pressure, and the calculation process is shown in equations (3.3)–(3.6). Solubility parameter component group contributions are shown in table 3.

3.3 $$\delta_{d}=\frac{\sum F_{\mathrm{di}}}{V},$$

3.4 $$\delta_{p}=\frac{\sum F_{\mathrm{pi}}}{V},$$

3.5 $$\delta_{h}=\sqrt{\frac{\sum E_{\mathrm{hi}}}{V}}$$

3.6 $$\;\mathrm{and}\;\delta =\sqrt{\delta_{d}^{2}+\delta_{p}^{2}+\delta_{h}^{2}},$$

where *δ* is the solubility parameter. *δ*_d_, *δ*_p_ and *δ*_h_ are the dispersion force, polarity force and hydrogen bonding force components of the solubility parameters, respectively, and the calculated values are 16.464, 0.07133 and 5.9000 MPa^1/2^. The solubility parameter of palmitic acid is 17.489 MPa^1/2^, which is similar to that calculated by molecular dynamics simulation, indicating the reliability of the simulation method.

**Table 3.** Solubility parameter component group contributions (Hoftyzer–Van Krevelen).

structural group	contributions		
	*F*_di_ ((MJ m^−3^)^1/2^ mol^−1^)	$F_{\mathrm{pi}}^{2}$ ((MJ m^−3^)^1/2^ mol^−1^)	*E*_hi_ (J mol^−1^)
−CH_3_	420	0	0
−CH_2_-	270	0	0
−COOH	530	420	10 000

**Figure 6.**
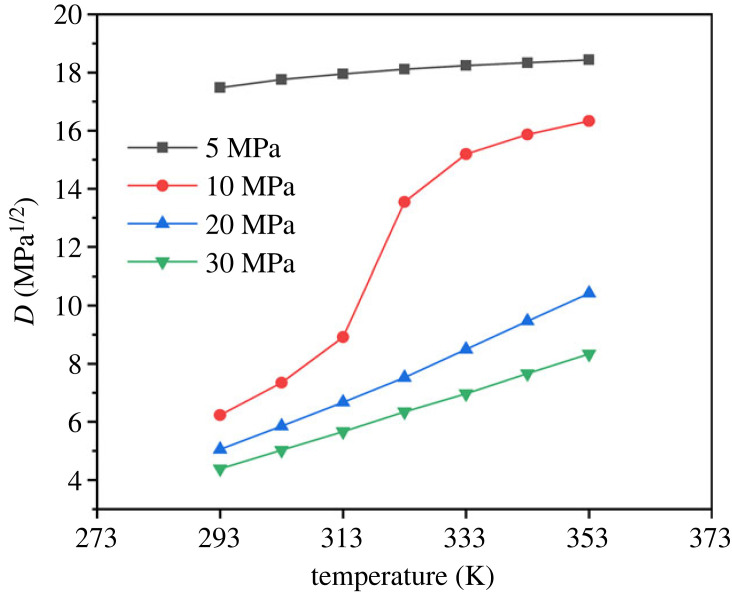
Solubility parameters difference between palmitic acid and CO_2_.

According to the principle of similar solubility parameters, the smaller the difference between the solubility parameters of palmitic acid and CO_2_, the better the solubility of palmitic acid in CO_2_. On the contrary, the larger the difference between the solubility parameters, the poorer the solubility of palmitic acid in CO_2_. The solubility parameter difference *D* was defined to be equal to the solubility parameter of palmitic acid minus the solubility parameter of CO_2_, and the solubility parameter difference under different temperature and pressure conditions is shown in figure 6, through which the solubility trend of palmitic acid with temperature and pressure could be intuitively seen. The difference of solubility parameters under 5 MPa is the largest and the influence of temperature is not obvious, which indicates that the solubility of palmitic acid in CO_2_ is extremely low under low pressure. The separation of fatty acids and their compounds from CO_2_ at low pressure, even without increasing the temperature, will be significant. In fact, the supercritical fluid extraction of oils and fats also adopts the depressurization separation method. Under the pressure of 10 MPa, the difference in solubility parameters changes significantly around 313 K, such as sharply increasing from 6.234 MPa^1/2^ at 293 K to 15.194 MPa^1/2^ at 333K, indicating the theoretical feasibility of isobaric extraction and separation. That is to say, liquid CO_2_ in the near critical zone is used for extraction, and the separation of fatty acids is achieved only by raising the temperature without changing the pressure. This will greatly overcome the huge energy consumption of the pressure reduction separation process of supercritical carbon dioxide extraction technology, which is also a major factor limiting the promotion of supercritical fluid technology.

**Figure 7.**
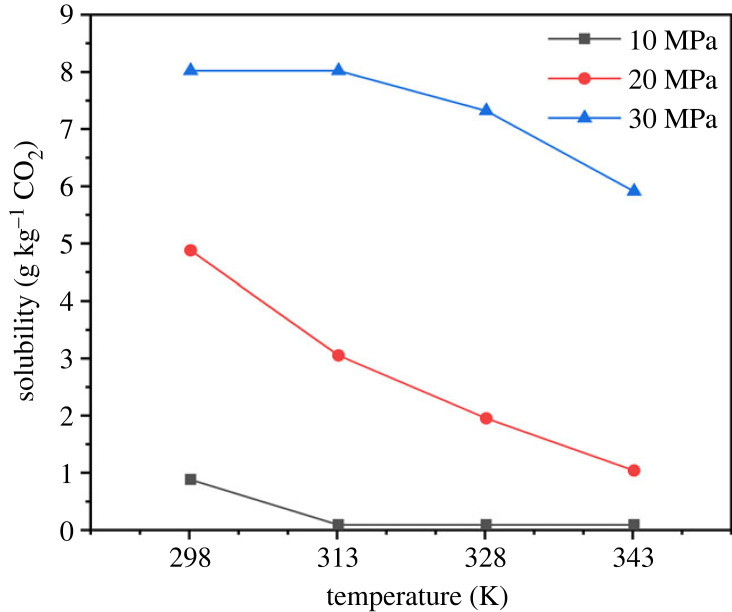
Solubility of canola oil in CO_2_.

Combined with the existing solubility data of canola oil in CO_2_ [27], as shown in figure 7, it can be found that the solubility of canola oil in liquid CO_2_ at 298 K is very high. Raising the temperature while maintaining the same pressure results in a significant change in solubility, especially at 20 MPa, indicating the possibility of oil extraction and separation under equal pressure. This is also consistent with the results of molecular dynamics simulation of the interaction between palmitic acid and CO_2_ to a certain extent. These all demonstrate the feasibility of using molecular dynamics simulation to guide the determination of the approximate range of process conditions and even the development of new processes. Molecular simulation technology will have a wider application in super/subcritical fluid extraction.

## 4. Conclusion

The molecular dynamics simulation method was used to construct pure CO_2_ system, pure palmitic acid system, and the mixed system of CO_2_ and palmitic acid in the range of 293–353 K, 5–30 MPa. The microscopic mechanism of the interaction between palmitic acid and CO_2_ was analysed. The error between the simulated density results of the pure CO_2_ system and the NIST database is small, and the error between the simulated solubility parameters of the pure palmitic acid system and the theoretical calculation results based on the group contribution method is also small, which proves the correctness of the simulation method. The snapshot of the micro distribution of palmitic acid under different temperatures and pressures shows that the molecular chain of palmitic acid is more straight and the molecules are more dispersed in high density CO_2_. Relatively, at low density, fatty acids undergo entanglement and aggregation. The radial distribution function further clearly shows that the dispersion of palmitic acid in CO_2_ gradually decreases with the increase of temperature, and increases with the increase of pressure. With the increase of temperature, the interaction energy between palmitic acid and CO_2_ decreases, and the solubility of palmitic acid decreases under the constraint of its own molecular force. With the increase of pressure, the interaction energy between palmitic acid and CO_2_ increases, which is conducive to overcoming the interaction between palmitic acid molecules and promoting dissolution. This is consistent with the solubility data of fatty acids reported in the literature and the setting rules of supercritical CO_2_ extraction process conditions. The solubility parameters of palmitic acid and CO_2_ can better reflect the trend of solubility changing with temperature and pressure. The solubility parameters of palmitic acid and CO_2_ vary sharply from 293 to 333 K at 10 MPa, providing theoretical support for the isobaric extraction and separation using liquid CO_2_ in the near critical region, and the existing solubility data of canola oil also provide experimental support. It can be seen that molecular simulation technology can play a guiding role in determining process conditions and developing new processes.

## Data Availability

Data are available from the Dryad Digital Repository: https://doi.org/10.5061/dryad.8sf7m0cv6 [28].
